# Complete Genome Sequence of a Soybean Dwarf Virus Isolate from White Clover in Germany

**DOI:** 10.1128/MRA.00637-20

**Published:** 2020-07-09

**Authors:** Yahya Z. A. Gaafar, Heiko Ziebell

**Affiliations:** a Institute for Epidemiology and Pathogen Diagnostics, Julius Kuehn Institute, Braunschweig, Germany; DOE Joint Genome Institute

## Abstract

In this study, we present the complete genome of a new isolate of soybean dwarf virus (SbDV) (genus *Luteovirus*, family *Luteoviridae*) from white clover in Germany. The complete genome of the isolate (JKI ID 23556) consists of 5,858 nucleotides and displays 94.98% nucleotide identity to its most similar SbDV relative (GenBank accession number MN412736).

## ANNOUNCEMENT

White clover (Trifolium repens L., family *Fabaceae*) is one of the most important pasture legumes, covering a wide range of climatic regions in the world (1). Its value is based on the high nutritional quality and digestibility of its herbaceous vegetation and the capability of atmospheric nitrogen fixation in the absence of fertilizer nitrogen (1). In 2007, a white clover plant sample showing symptoms indicative of a viral infection was collected in Braunschweig, Germany. The sample tested positive for soybean dwarf virus (SbDV) with an enzyme-linked immunosorbent assay (ELISA), as described previously (2); this was confirmed by reverse transcription (RT)-PCR using SbDV-specific primers (SbDVs and SbDVas) (2). The virus was transmitted by pea aphids (Acyrthosiphon pisum) to faba bean plants (Vicia faba L., family *Fabaceae*), and transmission was confirmed by both ELISA and RT-PCR. SbDV is a single-stranded positive-sense RNA virus and belongs to the genus *Luteovirus* (family *Luteoviridae*) (3). In Germany, white clover is known to be infected by SbDV (2).

To sequence the whole genome of SbDV JKI ID 23556, we followed the steps described previously (4). Briefly, total RNA was extracted from infected *V. faba* leaves using the innuPREP RNA minikit (Analytik Jena AG), and then rRNA was depleted with the RiboMinus plant kit (Invitrogen) following the manufacturers’ instructions. cDNA was synthesized using random octanucleotide primers and ProtoScript II reverse transcriptase (NEB), and second-strand cDNA synthesis was performed using the NEBNext Ultra II nondirectional RNA second-strand synthesis module kit (NEB) according to the manufacturer’s instructions. The libraries were prepared using the Nextera XT library kit (Illumina) and were sequenced on a MiSeq v3 platform (2 × 301 bp).

The raw reads (1,561,316 total reads) were analyzed using Geneious Prime v2020.1.2 software. The reads were quality trimmed (error probability limit, 0.05) and size filtered to >50 nucleotides. The complete sequence of JKI ID 23556 (5,858 nucleotides) was assembled according to the SbDV reference genome (GenBank accession number NC_003056) by mapping with the reference tool; 10,303 reads were mapped, with a mean coverage of 461×. The GC content of the complete genome of SbDV isolate JKI ID 23556 is 44.2%. A direct BLASTn search of the NCBI database showed that JKI ID 23556 shared the most similar nucleotide identity of 94.98% with SbDV GenBank accession number MN412736. A BLASTp search showed that the SbDV JKI ID 23556 proteins shared between 93.6% and 99.5% amino acid identities with their homologues from SbDVs in the NCBI database (Table 1). These results show that the sequences of all of the proteins of this isolate are most similar to the known sequences in GenBank but the movement protein is divergent. No other viruses were detected in the sequenced library using *de novo* assembly followed by BLASTn as described (4).

**TABLE 1:** Characteristics of proteins of SbDV isolate JKI ID 23556 and their most similar protein sequences in GenBank, determined using BLASTp

Protein	Length (amino acids)	Predicted mol wt (kDa)	Amino acid identity with most similar sequence (%)	GenBank accession no. for closest protein
RdRp P1-P2 fusion protein	894	102.1	98.3	AFP55353
RdRp P1 protein	361	41.6	97.2	NP_150431
Aphid transmission fusion protein	724	80.2	96.3	QJQ82512
Coat protein	200	22.2	99.5	ABR27529
Movement protein	189	21.4	93.6	QJQ82514

### Data availability.

The complete genome sequence of SbDV isolate JKI ID 23556 was deposited in GenBank under accession number MT543032. The raw data were deposited in the Sequence Read Archive (SRA) under BioProject accession number PRJNA524397, BioSample accession number SAMN15056231, and SRA accession number SRX8424802.
